# Expanding the Recessive Spectrum of Dilated Cardiomyopathy: RNA‐Level Validation of a Homozygous *CTNNA3* Splice‐Site Variant

**DOI:** 10.1155/humu/2079958

**Published:** 2026-07-18

**Authors:** Stefania Martino, Mara Doimo, Matteo Iacoviello, Caterina Giovine, Alessandro Stella, Cinzia Forleo, Rosanna Bagnulo, Gennaro Mariano Lenato, Andrea Igoren Guaricci, Eva Trevisson, Nicoletta Resta

**Affiliations:** ^1^ Medical Genetics Unit—Department of Preventive and Regenerative Medicine and Ionian Area (DiMePre-J), University Hospital Consortium Polyclinic of Bari, University of Bari Aldo Moro, Bari, Italy, uniba.it; ^2^ Clinical Genetics Unit, Department of Women′s and Children′s Health, University of Padova, Padova, Italy, unipd.it; ^3^ Pediatric Research Institute (IRP)—Fondazione Città della Speranza, Padova, Italy; ^4^ Medical Genetics, National Institute of Gastroenterology, IRCCS “Saverio de Bellis” Research Hospital, Castellana Grotte, Italy; ^5^ Cardiovascular Disease Section, Interdisciplinary Department of Medicine (DIM), University Hospital Consortium Polyclinic of Bari, University of Bari “Aldo Moro”, Bari, Italy, uniba.it; ^6^ Medical Genetics Unit, University Hospital Consortium Polyclinic of Bari, Bari, Italy

**Keywords:** *α*T-catenin, autosomal recessive cardiomyopathy, CTNNA3, DCM

## Abstract

CTNNA3 encodes *α*T‐catenin, an intercalated disc (ICD) protein essential for cardiomyocyte coupling. Human omics studies have shown reduced CTNNA3 expression, ICD ultrastructural disruption, and dilated cardiomyopathy (DCM)–associated hyperphosphorylation of *α*T‐catenin. Direct RNA‐level evidence linking biallelic CTNNA3 variants to human cardiomyopathy has been lacking. Clinical exome sequencing was performed in a 21‐year‐old man with DCM, severe left ventricular systolic dysfunction (LVEF 20%), and atrial fibrillation (AF). The identified homozygous variant (NM_013266.4 : c.1733 − 1G > C) was assessed with multiple splicing prediction tools and functionally validated via a minigene hybrid assay in HEK293 cells. Splicing predictors indicated loss of the canonical acceptor site. The minigene assay confirmed three aberrant transcripts: out‐of‐frame exon 13 skipping with a premature stop codon, a 24‐nucleotide in‐frame deletion, and partial intron retention, resulting in a possibly nonfunctional protein. Under guideline‐directed medical therapy, LVEF normalized, but a persistent arrhythmic phenotype remained, with recurrent AF, frequent ventricular ectopic beats, and nonsustained ventricular tachycardia. We report a recessive form of DCM associated with a homozygous canonical splice‐site variant in CTNNA3, encoding the ICD protein *α*T‐catenin. Our results are consistent with human omics studies. Altogether, these data provide evidence that biallelic CTNNA3 splice‐disrupting variants can cause human cardiomyopathy driven by ICD dysfunction. The dissociation between ventricular recovery and persistent arrhythmia highlights the complex phenotypic spectrum of CTNNA3‐related disease.

## 1. Introduction

Dilated cardiomyopathy (DCM) is defined by left‐ventricular dilatation with systolic dysfunction not fully accounted for by abnormal loading conditions or coronary artery disease. It is a major cause of heart failure, malignant arrhythmia, and heart transplantation. Current European guidance advocates a structured diagnostic pathway (family history, electrocardiogram [ECG], echocardiography, and cardiac MR), pre‐ and posttest counseling, and multidisciplinary interpretation. The identification of a pathogenic/likely pathogenic variant refines prognosis, may influence cardiac electronic device implantation, enables cascade testing, and, when relatives test negative, permits discharge from surveillance. In the absence of a molecular diagnosis, periodic clinical screening of first‐degree relatives is recommended [1, 2].

In the intercalated disc (ICD), *α*T‐catenin encoded by *CTNNA3* links the N‐cadherin/*β*‐catenin complex to actin and engages plakophilin‐2 within the mixed adherens–desmosomal “area composita,” thereby integrating mechanical adhesion with electrical coupling [3–5]. In vivo, *α*T‐catenin deficiency disrupts the area composita, reduces connexin‐43 at ICDs, and produces a DCM‐like phenotype with heightened susceptibility to ischemia‐provoked ventricular arrhythmias, underscoring a nonredundant role for *α*T‐catenin in junctional integrity and conduction [6]. Human myocardium studies are concordant. In fact, the failing left ventricle showed ICD ultrastructural remodeling with reduced *α*T‐catenin, whereas proteomics/phosphoproteomics revealed DCM‐associated *α*T‐catenin phosphorylation, mislocalization, and conduction impairment, implicating a *CTNNA3*‐centered pathway in the disease [7, 8].

Historically, *CTNNA3* mapped to 10q21 and was nominated as a DCM candidate, although early screens detected few clearly pathogenic coding variants consistent with marked locus and allelic heterogeneity [9]. Furthermore, two heterozygous variants of CTNNA3, a missense and an in‐frame deletion (c.281 T > A and c.2293_2295delTTG), were identified in two probands with arrhythmogenic right ventricular cardiomyopathy (ARVC), suggesting a possible association between CTNNA3 variations and ARVC [10]. Here, we describe a previously unreported recessive presentation: a homozygous canonical splice‐acceptor variant, NM_013266.4 (CTNNA3): c.1733 − 1G > C, in a proband with DCM. The variant is listed in ClinVar (Variation ID 541662) as a single submission of unknown significance. It occurs in population datasets at very low frequency (rs193155648; gnomAD v4.1.0 ≈ 0.002%, whereas no homozygous individuals are reported), supporting its rarity. Functional splicing analysis demonstrated multiple aberrant transcripts: the first one leading to a complete skipping of the 152‐bp exon with consequent frameshift/premature stop, a second causing an in‐frame 24‐bp deletion removing eight residues (VIPEFVTQ), and a third one resulting from an in‐frame partial intron retention, which encodes for a possibly nonfunctional protein. The results of functional analyses were precisely forecast by three different splicing prediction algorithms we used to survey the predicted effects of the mutated sequence. These findings dovetail with *α*T‐catenin biology at the ICD and may support a pathogenic role for CTNNA*3* due to a recessive human cardiomyopathy caused by abnormalities at the RNA‐level, reinforcing guideline‐directed, genotype‐informed family management in DCM [1, 7, 8].

## 2. Methods

### 2.1. Clinical Case

A 21‐year‐old man presented with dyspnoea and palpitations; a family history of sudden cardiac death was reported but could not be corroborated due to the absence of clinical documentation. He was admitted to the Cardiology Unit, Interdisciplinary Department of Medicine (DIM), University of Bari Aldo Moro, University Hospital Consortium, Polyclinic of Bari, Bari, Italy. His baseline ECG showed atrial fibrillation (AF) and low‐voltage QRS complexes. He was diagnosed with DCM with severe left ventricular systolic dysfunction (left ventricular ejection fraction [LVEF] = 20*%*), which showed good responsivity to optimized drug treatment for heart failure (last reported LVEF was 55%) [11, 12]. Coronary angiography revealed no abnormalities. Twenty‐four‐hour Holter monitoring demonstrated the presence of rare, monomorphic, and nonrepetitive ventricular premature beats. His AF was successfully reverted by electrical cardioversion.

In the following years, the patient had recurrent episodes of paroxysmal and persistent AF, which required multiple electrical cardioversions. The patient refused to undergo AF ablation. Long‐term oral anticoagulation therapy was initiated.

Seventeen years later, 24‐h ECG monitoring revealed persistent AF, accompanied by frequent polymorphic ventricular ectopic beats. This included 76 monomorphic couplets and 58 episodes of nonsustained ventricular tachycardia (NSVT), the longest of which lasted 12 beats, with a maximum ventricular rate of 196 bpm. He was admitted to our cardiology unit. ECG showed persistent AF, which was successfully reversed by electrical cardioversion. Coronary angiography revealed normal coronary arteries in terms of their origin, caliber, and course, with only mild, diffuse atherosclerosis present. Cardiac magnetic resonance (CMR) imaging documented no late gadolinium enhancement. The patient was recommended an implantable cardioverter‐defibrillator in primary prevention due to multiple NSVT episodes that were unresponsive to antiarrhythmic drugs, but he declined.

Seven years later, a follow‐up CMR scan confirmed a preserved global biventricular systolic function (LVEF 64%, right ventricular ejection fraction [RVEF] 61%). Quantitative analysis revealed no evidence of focal or diffuse myocardial fibrosis, inflammation, or regional wall motion abnormalities.

At the most recent evaluation, the patient′s ECG documented AF. A 24‐h Holter ECG revealed frequent (*n* = 2.033) monomorphic ventricular premature beats, but no sustained arrhythmia. An echocardiogram showed mild enlargement of the left atrium and mild concentric left ventricular hypertrophy, with preserved left ventricular systolic function (LVEF 55%).

### 2.2. Next‐Generation Sequencing and Library Preparation

The patient′s genomic DNA (gDNA) was extracted from peripheral blood stored in EDTA tubes using a QIAamp Mini kit (Qiagen, Hilden, Germany). Evaluation of gDNA concentration and quality was performed by using a Qubit dsDNA HS assay kit on a Qubit 2.0 fluorimeter (Invitrogen, Carlsbad, California, United States), according to the manufacturer′s instructions.

The patient′s gDNA was subjected to clinical exome sequencing (CES), with library preparation performed using the TruSight One Sequencing Panel (Illumina, San Diego, California, United States) and enrichment for regions of interest capture technology. Sequencing was performed on an Illumina NextSeq550Dx platform (Illumina, San Diego, California, United States), following the manufacturer′s instructions, using the NextSeq 550 System High‐Output kit.

Data analysis was performed using NextSeq Control Software v.4.2.0 and Local Run Manager Software v.4.0.0, both provided by Illumina. Reads were aligned to the human reference genome (GRCh37/hg19) using the BWA Aligner v.11.5 software [13]. Variant calling was performed using the Genome Analysis Toolkit (GATK) [14]. A mean coverage depth of 213x and a uniform coverage (Pct > 0.2^∗^mean) of 95.0% were achieved. Variant calling data were analyzed with Geneyx analysis software v.6.1.1 (Geneyx, Herzliya, Israel). Variants were filtered and prioritized using Human Phenotype Ontology (HPO) terms related to the patient′s cardiac phenotype [15], by means of in silico tools (Alamut Visual Plus Viewer [SOPHiA GENETICS, Lausanne, Switzerland]); Combined Annotation Dependent Depletion (CADD) v.1.6 [16], Geneyx analysis software, and public databases (Human Reference Genome hg19, dbSNP 138, OMIM [https://www.omim.org/], ClinVar [https://www.ncbi.nlm.nih.gov/clinvar] accessed on March 31, 2026; the Clinical Genome Resource [ClinGen], [https://clinicalgenome.org/] LOVD, and gene‐specific databases).

Variants were described in accordance with the nomenclature and recommendations of the Human Genome Variants Society (HGVS) [17] and classified according to the American College of Medical Genetics (ACMG) guidelines [18]. Furthermore, the detected variants were confirmed by Sanger sequencing on the SeqStudio Genetic Analyzer (Applied Biosystems, Waltham, Massachusetts, United States), according to the manufacturer′s instructions.

### 2.3. Single‐Nucleotide Polymorphism (SNP) Array Analysis

A high‐density SNP array investigation (Illumina Infinium CytoSNP 850K v1.4) was carried out, using the Infinium CytoSNP‐850K BeadChip (Illumina, Inc., San Diego, United States), which contains 848,902 selected SNPs spanning the genome with enriched coverage for 3262 genes of known relevance according to the manufacturer′s instructions. The data were analyzed using BlueFuseTM Multi v4.5 software (BlueGnome, BlueGnome Ltd. [now part of Illumina, Inc.], Cambridge, United Kingdom), with genomic positions presented as mapped to the human reference genome build (GRCh37/hg19). Accurate profiling of chromosomal aberrations, such as duplications/deletions, as well as copy‐neutral absence of heterozygosity events, was obtained. The significance of each of the detected variants was determined by comparing results with public databases of copy‐number variant (CNV), including the Database of Genomic Variants (DGV) (http://projects.tcag.ca/variation/ accessed on November 4, 2025), the Database of Chromosomal Imbalance and Phenotype in Humans Using Ensembl Resources (DECIPHER) (http://decipher.sanger.ac.uk/), the ClinGen (https://clinicalgenome.org/), and the UCSC Genome Bioinformatics database (http://genome.ucsc.edu). Variants were classified according to the ACMG guidelines [19] and were annotated according to the ISCN 2024 nomenclature [20].

### 2.4. In Vitro Minigene Hybrid Assay

The in vitro minigene hybrid assay was performed as described [21] using the *β*‐globin vector previously generated in the laboratory [22]. Briefly, an 873‐bp fragment including Exon 13 of the *CTNNA3* gene, part of the upstream intron, and part of the downstream intron was amplified from a healthy control gDNA. The fragment was cloned into the pcDNA3.1 *β*‐globin vector using the In‐Fusion HD Cloning Kit (Takara Bio, United States) according to the manufacturer′s instructions. To obtain the *CTNNA3* mutated allele (c.1733 − 1G > C), the QuikChange Lightning Site‐Directed Mutagenesis Kit (Agilent Technologies, United States) was used according to the manufacturer′s instructions.

The correct sequence of generated vectors was verified by direct Sanger sequencing. One clone containing the wild‐type allele and one with the mutation was retained for expression experiments.

HEK293 cells (3 × 10^5^) were transfected with 2 *μ*g of the wild type or the mutated minigene using Lipofectamine 2000 (Thermo Fisher Scientific, United States) according to the manufacturer′s instructions. After 48 h, total RNA was extracted using TRIzol reagent (Thermo Fisher Scientific, United States) and reverse transcribed with Superscript II reverse transcriptase (Thermo Fisher Scientific, United States). The resulting complementary DNA (cDNA) was then amplified using specific primers for the *β*‐globin gene. PCR products were separated on a 1% agarose gel and sequenced by Sanger sequencing using primers encompassing Exons 2 and 3.

The sequences of the oligonucleotides used in this study are listed in Table S1 (Table S1).

## 3. Results

### 3.1. Molecular Analysis

CES analysis did not identify any pathogenic or likely pathogenic variants in the genes currently associated with clinical indication; however, it revealed a homozygous variant of uncertain significance (VUS) in *CTNNA3,* NM_013266 : c.1733 − 1G > C. The patient′s parents′ gDNA was unavailable for genetic testing, which prevented us from confirming a biparental inheritance of the identified *CTNNA3* variant and thus true homozygosity in the proband. To better characterize the zygosity of the c.1733 − 1G > C variant and to rule out potential experimental mimics, such as the presence of a CTNNA3‐involving deletion‐associated hemizygosity or an allele dropout, we assessed the genomic profile of the whole region through high‐density SNP‐array.

SNP‐array analysis showed a normal disomic profile, which found no evidence of CNV at the CTNNA3 locus. Furthermore, it was identified a 157‐Mb short stretch of homozygosity on 10q21, encompassing the 3 ^′^ portion of the *CTNNA3* gene and several adjacent genes, located toward the centromeric direction. Since the variant NC_000010.10 : g.68040380C > G (NM_013266.4 c.1733 − 1G > C) falls within this homozygosity stretch, SNP array–based results support true homozygosity and biparental, identity‐by‐descent inheritance of the c.1733 − 1G > C variant, rather than hemizygosity or allele dropout. Subsequently, segregation analysis was performed on the healthy 49‐year‐old sister′s proband in whom the CTNNA3 variant was detected in a heterozygous state.

### 3.2. Bioinformatic Analyses

To predict the variant effect on the splicing, the sequence surrounding the variant NM_013266.4 (CTNNA3): c.1733 − 1G > C was inspected with four different algorithms: MaxEntScan [23], Pangolin [24], SpliceAI [25], and SPiP [26].

**Figure 1:**
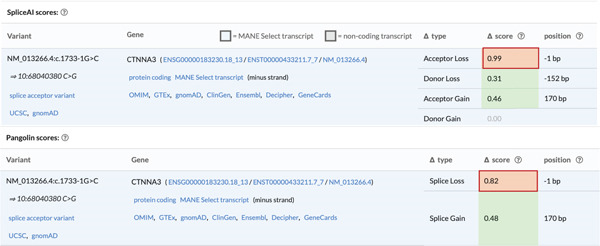
In silico splice predictions: Both Splice AI and Pangolin predict the wild‐type acceptor loss (highlighted in orange) and the creation of a novel acceptor site 170‐bp upstream from the wild‐type splice acceptor site, which was fully recapitulated from the in vitro minigene analysis.

MaxEntScan predicted a decrease of the 3 ^′^ splicing site score from 6.54 to −1.53, with an expected decrement ratio of normal splicing equal to −123.39%. Both SpliceAI and Pangolin forecast the wild‐type acceptor site loss and the creation of a novel acceptor site, as detailed in Figure 1. Finally, SPiP predicted a significant alteration of the consensus splice site, with an overall risk estimate of 98.41% (range 91.47%–99.96%) for the variant to alter splicing.

### 3.3. Effects of Variant **CTNNA**3 **c**.1733 − 1**G** > **C** on the Splicing Mechanism

To test the NM_013266.4(CTNNA3): c.1733 − 1G > C variant effect on splicing, we performed an in vitro minigene hybrid assay [21].

**Figure 2:**
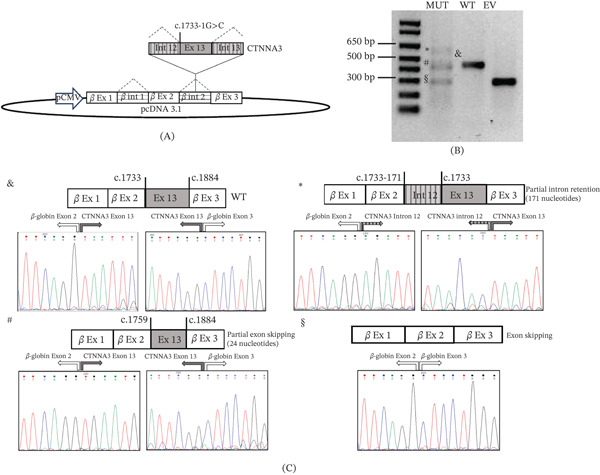
The in vitro minigene hybrid assay results: (A) schematic representation of the hybrid minigene construct used in the experiment. *β*‐globin exons and introns are depicted in white, respectively, whereas introns and exon 13 of CTNNA3 are depicted in grey. (B) Agarose gel electrophoresis of RT‐PCR products obtained from transfection of HEK293 cells with the minigene construct harboring the variant c.1733 − 1G > C in the CTNNA3 gene and the corresponding wild type (WT). Empty vector (EV) was used as internal control. (C) Schematic representation of the transcripts′ structure obtained from the hybrid minigenes. Symbols ∗, #, §, and & identify the respective bands in (B). Corresponding Sanger sequencing of the RT‐PCR products are depicted below. Variant c.1733 − 1G > C causes the accumulation of three aberrant splicing products: the lowest one (§) characterized by complete exon skipping, a middle one (#) characterized by partial skipping of 24 nucleotides, and the highest one (∗) that contains the retention of part of the Intron 12 (171 nucleotides).

The genomic region encompassing the exon and the surrounding intronic regions, including the variant of interest, was cloned into a vector for expression in mammalian cells. The final assembled vector carried Exons 1, 2, and 3 of the *β*‐globin gene and respective introns (Figure 2A).

Expression analysis of the hybrid minigene in HEK cells by reverse‐transcription PCR (RT‐PCR) showed that variant c.1733 − 1G > C had a significant effect on splicing, generating three different transcribed products (Figure 2C), as shown by gel electrophoresis (Figure 2B).

Sanger sequencing of three RT‐PCR bands revealed that the variant affects the splicing of exon 13 with different mechanisms (Figure 2): by causing the skipping of the entire exon, resulting in a frameshift and the introduction of a premature stop codon (lower band), by activating a cryptic exonic splice site leading to an in‐frame deletion of 24 nucleotides (middle band), and finally, by causing a partial in‐frame intron insertion of 171 nucleotides (upper band) (Figure 2B). No residual wild type transcript was detected in our in vitro assay.

These results support a deleterious effect of the variant on gene expression.

The in vitro minigene analysis fully recapitulated the bioinformatic analysis from SpliceAI and Pangolin, demonstrating that the variant c.1733 − 1G > C causes the absence of the normally spliced transcript and the concurrent presence of an aberrant transcript, which included 171 nucleotides downstream of the novel acceptor. In addition, the minigene assay allowed for the identification of two additional aberrant transcripts, one with a premature stop codon and another with an in‐frame inclusion of 24 additional nucleotides.

## 4. Discussion

This study expands the genetic spectrum of DCM by providing RNA‐level evidence that a homozygous canonical splice‐site variant in *CTNNA3* (NM_013266.4 : c.1733 − 1G > C) underlies a recessive form of disease. Although recessive inheritance has been historically considered uncommon, it represents clinically relevant and probably underrecognized architecture in DCM. These observations underscore the increasing genetic heterogeneity and the complex interplay between dominant and recessive mechanisms in inheritable cardiac conditions, with direct implications for the design, interpretation, and equitable application of clinical genetic testing.

Although *CTNNA3* has long been considered a biologically plausible DCM candidate based on its role at the ICD, robust human genotype–phenotype correlations remain scarce and are largely limited to heterozygous variants [10]. In this context, our work provides new evidence for *CTNNA3* in the etiology of DCM by (i) documenting a recessive presentation with a canonical acceptor‐site variant in a young adult with DCM; (ii) demonstrating, through a functional minigene assay, a profound disruption of splicing with multiple aberrant transcripts fully consistent with in silico predictions; and (iii) integrating these findings with animal models and omics data that converge on *α*T‐catenin as a nonredundant node in mechanoelectrical coupling at the ICD.

The c.1733 − 1G > C variant affects the −1 position of the Exon 13 acceptor site, a location typically associated with a high prior probability of pathogenicity. Consistent with this, four independent splicing prediction tools (MaxEntScan, SpliceAI, Pangolin, and SPiP) uniformly forecast abolition of the native acceptor site and activation of an upstream cryptic site, with a very high predicted probability of splicing disruption. These predictions were not merely suggestive but mechanistically precise: Both SpliceAI and Pangolin indicated loss of the wild‐type acceptor and the creation of a novel upstream acceptor: an architecture faithfully recapitulated in the minigene assay (Figure 2).

The in vitro splicing analysis showed that the variant generates at least three aberrant transcripts, each highly likely to compromise *α*T‐catenin function, either through nonsense‐mediated decay of truncated transcripts or via structurally altered proteins with disrupted domain architecture and/or interactions at the ICD. Importantly, the normally spliced product was absent, supporting a loss‐of‐function mechanism rather than a subtle splicing modulation.

Although parental DNA was unavailable, preventing formal demonstration of biparental inheritance, several lines of genomic evidence strongly support true homozygosity. Moreover, the proband′s 49‐year‐old sister carries the variant in a heterozygous state, with no evidence of cardiac clinical manifestations, reinforcing our findings.

SNP‐array data excluded CNVs on chromosome 10q and identified a 1.57‐Mb region of homozygosity encompassing CTNNA3, consistent with segmental identity‐by‐descent rather than allele dropout or hemizygosity. The variant is extremely rare in population databases and is exclusively observed in the heterozygous state, consistent with a recessive model.

Our clinical observations closely mirror the phenotype reported in *α*T‐catenin–deficient murine models, in which disruption of the area composita leads to a DCM‐like cardiomyopathy with conduction abnormalities and increased susceptibility to ventricular arrhythmias. In these models, loss of *α*T‐catenin destabilizes the N‐cadherin/*β*‐catenin complex, decreases connexin‐43 at the ICD, and alters mechanical and electrical coupling. The proband′s presentation with LV dilatation and severe systolic dysfunction in early adulthood, in the absence of coronary artery disease or overt myocardial fibrosis, is in line with a primary cardiomyopathic process rather than secondary remodeling. Equally notable is the cardiac rhythm profile: In mice, *α*T‐catenin loss predisposes to ventricular arrhythmias, particularly under ischemic or stress conditions, and in our patient, frequent ventricular ectopy and episodes of NSVT occurred despite normalization of LV ejection fraction under guideline‐directed medical therapy. This suggests that the arrhythmic substrate may at least partly reflect primary junctional and conduction abnormalities rather than simply the degree of systolic dysfunction. The long‐standing burden of AF, beginning at a relatively young age and recurring despite cardioversion, further supports the concept that *CTNNA3* dysfunction affects atrial as well as ventricular conduction, in keeping with the widespread presence of the area composita at atrial–ventricular junctions.

However, there are important differences compared with animal models that may elucidate pathophysiological mechanisms. Whereas *α*T‐catenin knockout mice often show progressive ventricular deterioration, our patient experienced near‐complete recovery of LV function and stable preservation of biventricular systolic performance on long‐term follow‐up. Possible explanations include incomplete loss of functional protein due to residual spliced transcripts predicted to preserve the frame, compensatory upregulation of other *α*‐catenin isoforms or junctional components, and the impact of early initiation of disease‐modifying heart failure therapy. These observations suggest that CTNNA3‐related cardiomyopathy in humans may manifest not only as a relentless progressive DCM but also as a dynamic phenotype with partial reversibility of systolic dysfunction, whereas arrhythmic vulnerability persists.

Proteomic and phosphoproteomic analyses of failing human myocardium have repeatedly implicated CTNNA3 and the area composita in the broader DCM network, revealing reduced *α*T‐catenin abundance, altered phosphorylation states, and mislocalization associated with conduction slowing and electrical uncoupling. These omics datasets position CTNNA3 at the intersection of mechanical adhesion, cytoskeletal organization, and ion‐channel distribution; however, to date, they lack a well‐characterized, functionally validated loss‐of‐function variant in humans as an anchor for causal inference. Results in our proband might link this omics‐derived CTNNA3‐centered network to a clinically manifest cardiomyopathy and arrhythmic phenotype. Moreover, our findings support a model in which *α*T‐catenin deficiency creates a vulnerable substrate characterized by altered intercellular coupling and conduction heterogeneity.

From a translational standpoint, the convergence between monogenic human data and system‐level omics has two main implications. First, it elevates *CTNNA3* from a “candidate” gene to one in which rare, biallelic, pathogenic variants can be sufficient to cause disease, justifying its inclusion and careful interpretation in cardiomyopathy gene panels. Second, it strengthens the rationale for exploring junctional and conduction‐related biomarkers or targets in DCM subsets with ICD involvement.

Notably, the patient declined ICD implantation despite recurrent NSVT episodes unresponsive to antiarrhythmic drugs. Although LV function recovered, the persistent arrhythmic burden and the mechanistic link between *α*T‐catenin deficiency and conduction abnormalities argue for a relatively low threshold for device consideration in similar genotypes.

This work integrates high‐depth NGS, SNP‐array analysis, multiple splicing prediction algorithms, and a minigene functional assay to build a coherent and biologically plausible narrative from DNA variant to RNA defect to clinical phenotype. The use of an in vitro hybrid minigene allowed precise dissection of splicing outcomes and direct comparison between wild type and mutant alleles under controlled conditions. The concordance between computational predictions and experimental results further supports the utility of modern splicing prediction tools as a triage strategy for intronic and splice‐site variants in cardiomyopathy genes.

Some limitations should be acknowledged. First, this is a single case, and the absence of parental and extended family genotyping precludes formal segregation analysis. Second, the minigene assay, although powerful, employs HEK293 cells rather than cardiomyocytes; cell type–specific splicing factors could, in principle, modulate the relative abundance of the aberrant isoforms in the human heart. In fact, myocardial tissue from the proband was not available, preventing direct assessment of *α*T‐catenin expression, localization, and ICD ultrastructure. Third, we could not evaluate CTNNA3 mRNA alterations directly in a whole blood tissue sample, where this gene is virtually not expressed (median TPM = 0.004950 in males; GTEx portal: https://gtexportal.org/home/gene/CTNNA3/geneExpressionTab). Also, we cannot exclude the contribution of additional genetic or environmental modifiers, although no other clearly pathogenic variants were identified in known cardiomyopathy genes.

This case opens several avenues for future research and clinical translation. At the genomic level, larger mechanisms and population‐based sequencing studies are needed to define the prevalence and spectrum of *CTNNA3* loss‐of‐function variants, clarify the relative contributions of recessive versus dominant mechanisms, and delineate the range of cardiac phenotypes—from isolated arrhythmia to overt DCM.

Finally, our findings reinforce the importance of maintaining *CTNNA3* within comprehensive cardiomyopathy gene panels and the utility of coupling NGS with functional assays to assess the pathogenicity of rare splice‐site variants precisely. In families with otherwise unexplained DCM and arrhythmia, particularly when recessive inheritance patterns or regions of homozygosity are present, *CTNNA3* should be considered a candidate gene for cardiac‐related phenotypes. In the future, as more cases accumulate, the integration of clinical, imaging, omics, and electrophysiological data will be essential to build a genotype‐informed framework for prognosis and tailored management in CTNNA3‐related cardiomyopathy.

In summary, the homozygous CTNNA3 c.1733 − 1G > C splice‐site variant, identified in our proband, supports the hypothesis of a putative role of *α*T‐catenin loss of function in human cardiomyopathy, consistent with data from animal models and omics‐derived ICD networks.

## Author Contributions

N.R. supervised and coordinated the project. M.I., C.F., and A.I.G. collected clinical data. R.B., M.D., C.G., and S.M. acquired experimental data. S.M., A.S., M.D., G.M.L., E.T., and N.R. interpreted the results. S.M., M.D., M.I., C.F., E.T., and N.R. wrote the manuscript. All authors revised the manuscript draft. S.M. and M.D. have contributed to the work equally and should be regarded as co‐first authors.

## Funding

This study was supported by the European Union–Next Generation EU–NRRP M6C2 (PNRR‐TR1‐2023‐12377161, CUP I93C24000190006), Fondazione Città della Speranza (10.13039/100007363) (IRP 24/09), PNRR‐Young Researcher MSCA program, Italian Ministry of University and Research and Fondazione Città della Speranza (IRP‐StG‐2024), and Ministero della Salute (10.13039/501100003196) (GENERA: T3‐AN‐04). Open access publishing was facilitated by the Universita degli Studi di Bari Aldo Moro, as part of the Wiley‐CRUI‐CARE agreement.

## Disclosure

All authors approved the final version of the manuscript.

## Ethics Statement

The samples were obtained with appropriate informed consent signed by the patient in accordance with the ethical standards laid down in the 1964 Declaration of Helsinki and its later amendments. Informed consent was obtained from the patient to publish clinical and genetic information. The clinical risk management unit of the Policlinico of Bari has approved the consent form signed by our patients.

## Conflicts of Interest

The authors declare no conflicts of interest.

## Supporting information


**Supporting Information** Additional supporting information can be found online in the Supporting Information section. Table S1: Sequences of the oligonucleotides used in the in vitro minigene hybrid assay, including primers for amplification of the CTNNA3 exon 13 genomic fragment, for introduction of the c.1733 − 1G > C mutation by site‐directed mutagenesis, and for amplification and Sanger sequencing of the cDNA derived from the *β*‐globin vector (Exons 2 and 3).

## Data Availability

The data that support the findings of this study are available from the corresponding author upon reasonable request.
