# Supplementary material for: Expanding the Recessive Spectrum of Dilated Cardiomyopathy: RNA‐Level Validation of a Homozygous CTNNA3 Splice‐Site Variant
Source: Hum Mutat. 2026 Jul 18;2026:2079958. doi: 10.1155/humu/2079958 (PMC13379898; doi:10.1155/humu/2079958)
Supplement: Supplementary file 1 — Supporting Information Additional supporting information can be found online in the Supporting Information section. Table S1: Sequences of the oligonucleotides used in the in vitro minigene hybrid assay, including primers for amplification of the CTNNA3 exon 13 genomic fragment, for introduction of the c.1733 − 1G > C mutation by site‐directed mutagenesis, and for amplification and Sanger sequencing of the cDNA derived from the β‐globin vector (Exons 2 and 3). [file HUMU-2026-2079958-s001.pdf]

Supplementary Table 1:
Oligonucleotides used in this study

	Name	Sequence (5'-3')
**Vector amplification**	**pCDNA3.1_beta-globin_Infusion_F**	CTTGTACACATATTGACCAAATC
	**pCDNA3.1_beta-globin_Infusion_R**	ACATATTAAAACATTACACTTTAAC
**Insert amplification**	**CTNNA3_Infusion_F**	AATGTTTTAATATGTGAGAAATGATTGTATGGTCCCAAGG
	**CTNNA3_Infusion_R**	CAATATGTGTACAAGTCCTATCAATTCCACTACAGTGA
**Mutagenesis**	**CTNNA3_g2213c_For**	GACAATCTTCTCCTTATTCTTTATTTTAACACTAATTCCTGAATTTGTAACACA
	**CTNNA3_g2213c_Rev**	TGTGTTACAAATTCAGGAATTAGTGTTAAAATAAAGAATAAGGAGAAGATTGTC
**Screening**	**beta-globEX2_F**	TTGAGTCCTTTGGGGATCTG
	**beta-globEX3_R**	ACCAGCCACCACTTTCTGAT
